# Early childhood caries (ECC) prediction models using Machine Learning

**DOI:** 10.4317/jced.61514

**Published:** 2024-12-01

**Authors:** Daniel José Blanco-Victorio, Roxana Patricia López-Ramos, Johan Daniel Blanco-Rodriguez, Nieves Asteria López-Luján, Gina Fiorella León-Untiveros, Ana Lucy Siccha-Macassi

**Affiliations:** 1 Facultad de Ciencias e Ingeniería Universidad Peruana Cayetano Heredia Lima. Perú; 2 Universidad Científica del Sur. Lima. Perú; 3 Facultad de Ingeniería, Escuela de Ingeniería de Sistemas, Universidad de Lima. Perú; 4 Hospital Santa María del Socorro, Ica-Perú; 5 Facultad de Ciencias de la Salud, Universidad Nacional del Callao. Perú; 6 Faculty of Sciencies of Health. Universidad Nacional del Callao

## Abstract

**Background:**

To evaluate the performance of different prediction models based on machine learning to predict the presence of early childhood caries.

**Material and Methods:**

Cross-sectional analytical study. The sociodemographic and clinical data used came from a sample of 186 children aged 3 to 6 years and their respective parents or guardians treated at a Hospital in Ica, Peru. The database with significant variables was loaded into the Orange Data Mining software to be processed with different prediction models based on Machine Learning. To evaluate the performance of the prediction models, the following indicators were used: precision, recall, F1-score and accuracy. The discriminatory power of the model was determined by the value of the ROC curve.

**Results:**

76.88% of the children evaluated had cavities. The Support Vector Machine (SVM) and Neural Network (NN) models obtained the best performance values, showing similar values of accuracy, F1-score and recall (0.927, 0.950 and 0.974; respectively). The probability of correctly distinguishing a child with ECC was 90.40% for the SVM model and 86.68% for the NN model.

**Conclusions:**

The Machine Learning-based caries prediction models with the best performance were Support Vector Machine (SVM) and Neural Networks (NN).

** Key words:**Early childhood caries, Caries prediction, Machine Learning, Artificial intelligence, caries.

## Introduction

Dental caries is a public health issue that affects every nation in the world and has a significant impact on the health of people regardless of their social status, economic situation or age. It has been recognised by the World Health Organisation (WHO) as a public health problem due to its ubiquity and potential for acute complications (1). This chronic condition is characterised by the gradual degradation of the hard tissues of the tooth, mainly due to the metabolic activities of bacteria settled in the oral cavity (2). The demineralisation of tooth enamel and dentine occurs as a result of acids produced by bacteria permanently settled on the biofilm plaque of the teeth. The factors involved in the development of dental caries are diverse, including biological factors such as microbial presence, dietary habits, income, hygiene habits, age, etc.(2)

Artificial Intelligence (AI) is a general term used to describe the theory and development of computer systems that can perform tasks that normally require human cognition. Among the cognitive abilities that AI tries to mimic are: perception, comprehension, language, learning, planning, reasoning and problem solving. In healthcare, the use of AI is still restricted to specific and limited tasks as human cognition is still necessary for decision-making. This is because AI lacks the more complex skills that are inherent to human intelligence, such as the ability to handle a wide diversity of data sources and the experience that comes from years of activity. Nor does AI take into account factors external to its system, such as patient expectations (3). One of the modalities of AI is Machine Learning (ML), in which computers learn to find underlying rules from a set of data provided by the user, rather than humans providing these rules.

Basically, machine learning (ML) “learns” intrinsic statistical patterns in observed data (training data) to make predictions about unseen data (test data) (3). This learning from data by capturing its intrinsic statistical patterns and structures can be done in three ways: Supervised learning in which data and data labels (output) are provided, and the ML model is iteratively optimised to represent this data-label pair; Unsupervised learning which is used when the data provided to the learning algorithm is unlabelled and the algorithm is asked to identify patterns in the input data; Deep learning which are mainly used to process large and complex image data with the aim of classifying them by extracting image features such as edges, corners, shapes and macroscopic patterns using layers of filters (4).

In dentistry, AI has been used mainly to obtain more accurate diagnoses in a more efficient and faster way in order to obtain better treatment outcomes. The integration of machine learning and traditional image processing in dentistry has led to many applications, such as the automatic identification and numbering of teeth, caries, anomalies, disease detection based mainly on radiological images (5-10), to detect and classify periodontal diseases (11,12), to evaluate the performance of prosthodontic implants (13,14); to diagnose, plan and place landmarks for orthodontic treatments (15,16).

AI-based applications streamline care, freeing dental health professionals from laborious routine tasks, decreasing the costs involved in dental treatments for a wider population, and ultimately facilitating personalised, predictive, preventive and participatory dentistry. However, the solutions that AI provides have largely not entered routine dental practice, mainly due to the limited availability and accessibility of data, the lack of methodological rigour and standards in their development, and practical questions around the value and utility of these solutions (3). The aim of this research is to compare the performance of different prediction models based on machine learning to predict the presence or absence of early childhood caries.

## Material and Methods

Analytical study. The sociodemographic and clinical data used came from a sample of 186 children aged 3 to 6 years and their respective parents or guardians attended at the Dental Service of the Hospital Santa María del Socorro in Ica, Peru, in the 2023. The present study has the approval of the hospital Ethics Committee with N° 2023-100-16.

The clinical oral examination to determine the presence or absence of ECC was performed by two specialists trained in the ICDAS II assessment system (Kappa concordance test > 0.85). Intraoral clinical examinations were performed according to WHO recommendations using a flat mouth mirror N°5 and periodontal probes. The collection of the blood sample for haemoglobin dosage was performed by finger prick. In addition, parents completed a questionnaire to obtain information on the variables considered in the study. The following information was collected: age and sex of the child, consumption of sweets, haemoglobin level, type of delivery, birth order; age, sex, marital status, level of education, number of (living) children of the mother/guardian; monthly family income.

The data were pre-processed to exclude variables that did not contribute significantly to explaining the variable of interest. The Mann-Whitney-Wilcoxon U-tests were used for continuous variables and chi-square for categorical variables (*p-value*<0.05), leaving the following variables with significant contribution: age, family income level, haemoglobin level in blood, frequency of brushing, consumption of sweets and parents’ level of education. The database with the latter variables was loaded into Orange Data Mining software to be processed with different prediction models based on Machine Learning. Seventy percent of the data were used as training data and the other 30% as test data. The prediction models used were: Random Forest (RF), Gradient Boosting Decision Tree (GBDT), Support Vector Machine (SVM), Logistic Regression (LR), Neural Networks (ANN), K-Nearest Neighbours (k-NN).

Logistic regression is a linear algorithm used to predict the probability of occurrence of a dichotomous (two possible values) target or dependent variable. This means that there are only two possible outcomes. Extreme Gradient Boosting (EGB) is an algorithm based on decision tree diagrams and shows the same behaviour as standard linear regression. Random Forest is a linear combination of decision trees that creates decision trees on samples of data and then derives the prediction from each of them to, in the end, select the best solution by simple voting. Random Forest is a method that works on a subset basis and is better than a single decision tree, as it reduces overfitting by averaging the results. A decision tree is a prediction modelling technique. This technique applies a predictive model to go from observations about an element represented in the branches to conclusions about the target value shown in the leaves. K-Nearest Neighbours is a non-parametric classification technique in which the input consists of the k nearest training examples from the data set. Support Vector Machine is a linear classification model that can solve linear and non-linear problems. In essence, SVM is an algorithm that takes data as input and classifies it, if possible, using a line or hyperplanes (17).

**Figure 1:**
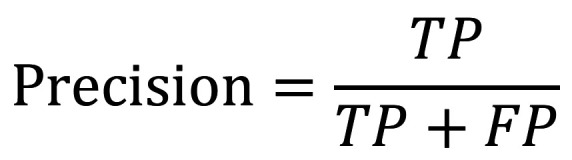
Formula.

The following indicators were used to assess the performance of the prediction models:

Precision: It is defined as the ability to classify or identify a datum as a true positive (TP) when a positive observation is also predicted to be positive. A false positive (FP) is a negative observation that is indicated as positive. It attempts to answer the question: ¿What proportion of positive identifications were correctly predicted as positive?, (Fig. 1).

**Figure 2:**
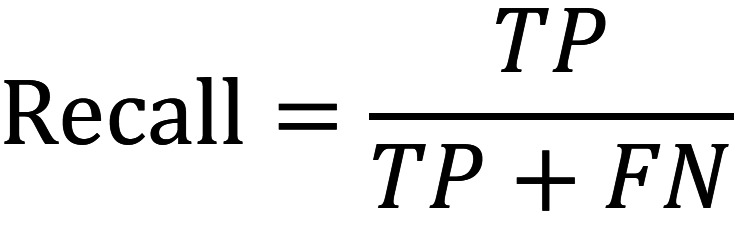
Formula.

Recall (Exhaustiveness/Sensitivity): Indicates the proportion or rate of true positives identified as such compared to the total number of true values (TP and FN correctly identified) predicted by the model. FN (false negative) represents a positive observation that was predicted as negative, (Fig. 2).

**Figure 3:**
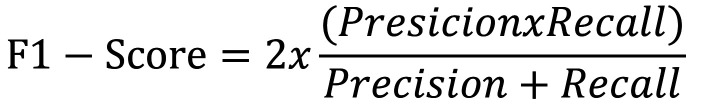
Formula.

F1-Score: It is a combination of the Precision and Recall measures. It is the weighted average of Precision and Recall. A high value is obtained only if the values of Precision and Recall are also high, (Fig. 3).

**Figure 4:**
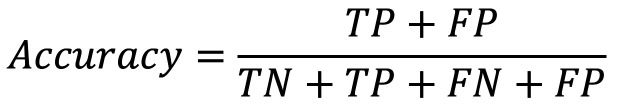
Formula.

Accuracy: Determines the efficiency of a model in identifying patterns and relationships between variables in a data set. It is the ratio of correctly predicted values to the total number of assessments, where TN (true negative) is the negative observation correctly predicted as negative, (Fig. 4).

**Figure 5:**
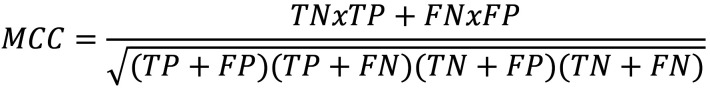
Formula.

Matthews correlation coefficient: It takes into account true and false positives and negatives and is generally considered a balanced measure that can be used even if the classes are of very different sizes, (Fig. 5).

The ROC curve, a graph showing the sensitivity of the model as a function of its specificity, was used to graphically evaluate the discriminative ability of the models evaluated.

The software used to perform the predictive model comparison was Orange Data Mining. Orange (http://orange.biolab.si) is a free, general-purpose machine learning and data mining tool. Its multi-layered architecture is suitable for different types of users, from data mining beginners to programmers who prefer a scrip interface. It can be used through Python scripting or with visual programming using GUI components called widgets (18). Data analysis in Orange data mining is implemented through workflows in a powerful and easy-to-use visual programming environment. Orange includes a number of techniques, such as data management and pre-processing, supervised and unsupervised learning, performance analysis, and a range of data visualisation and modelling techniques (19).

## Results

186 preschoolers treated at the de Hospital Santa María del Socorro in Ica, Peru, participated in the present study. In the final assessment,  Table 1 shows that 76.88% (143) of the children had ECC. In contrast, 23.12% (43) had no ECC.

**Figure 6:**
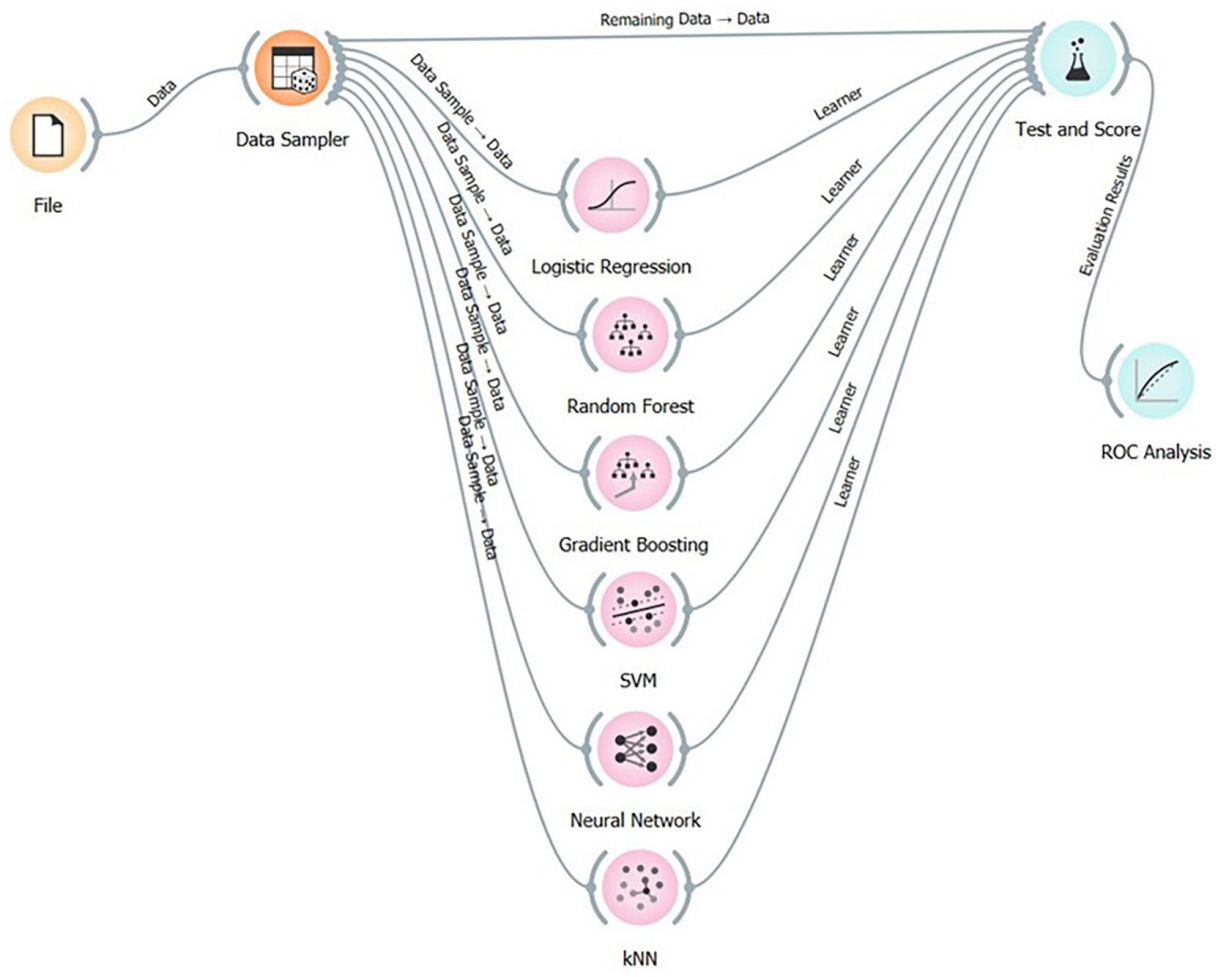
Workflow in Orange Data Mining with the different prediction models evaluated.

In adittion, Figure 6 shows the workflow used in Orange Data Mining with the different prediction models evaluated. The widgets used were: File, to load the database; Data Sampler, to divide the database into two groups (70% for training, 30% for testing); Test and score, to obtain the values that indicate the performance of the model; and ROC Analysis, to visualise the discriminative ability of the evaluated models.

**Figure 7:**
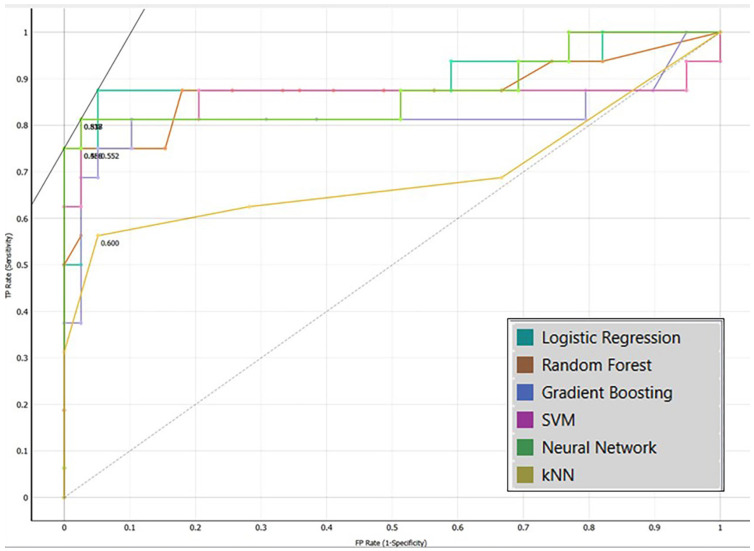
ROC curve of the models evaluated.

Table 2 shows the performance indicators of the models evaluated. The SVM and Neural Network models obtained the best performance values, showing accuracy, F1-score, precision, recall and Matthews correlation coefficient values of 0.927, 0.950, 0.927, 0.974 and 0.820; respectively. Although the above values are equal, the area under the ROC curve (AUC) varies, with the Neural Network model having the highest discrimination or classification power. The Neural Network model and the SVM model have a 90.40% and 86.68% probability of distinguishing between a child with ECC and without ECC, respectively. However, both are outperformed in discriminatory power by the logistic regression model which has a 91.10% probability of distinguishing between a child with ECC and without ECC. This can be visualised in Figure 7 which shows the ROC curve of the models evaluated.

Discussion

Early childhood caries (ECC) is one of the most common dental diseases in children and is a public health problem that has been increasing in recent times. If not treated in the early stages it can reach the deep layers of tooth structure involving the dental pulp and causing pain and infection (20). Therefore, timely and accurate diagnosis for the prevention and treatment of early childhood caries (ECC) is very important to preserve the future oral health of patients. Determining caries risk is an important part of a dental practice and various models have been developed to determine risk factors.

AI has recently been used in the field of dental health. Due to the possibility of storing large amounts of data and the existence of models based on Machine Learning and Deep Learning, it is now possible to generate algorithms that can detect pathologies, establish diagnoses and propose treatments with high success rates (21). AI has been used in different dental specialties such as periodontics, orthodontics, implantology, forensic dentistry, oral pathology and diagnosis (22). The aim of this study was to compare the performance of different prediction models based on machine learning to predict the presence or absence of ECC.

In the final evaluation, 76.88% of the children evaluated were found to have ECC; this prevalence is lower than that found by Pessaresi *et al*. (23) in suburban Lima (91.2%) and that reported by MINSA (85%) (24). It is higher than that found by Falcon-Aguilar (25) who found a prevalence of only 9.8% in children treated at the Cayetano Heredia National Hospital in Lima; it is also higher than that found by Díaz (26) who found a prevalence of 50% at the same facility. It coincides with a study conducted with children from Ecuador, Nepal and Vietnam that found a prevalence of 72% and with Mei *et al*. (27) whose found a prevalence of 76.4% in children from China.

The present study found that the SVM and Neural Network models obtained the best performance values, showing accuracy, F1-score, precision, recall and Matthews correlation coefficient values of 0.927, 0.950, 0.927, 0.974 and 0.820; respectively. Although the above values are equal, the area under the ROC curve (AUC) varies, with the Neural Network model having the highest discrimination or classification power. The Neural Network model and the SVM model have a 90.40% and 86.68% probability of distinguishing between a child with ECC and without ECC, respectively. However, both are surpassed in discriminatory power by the logistic regression model which has a 91.10% probability of distinguishing between a child with ECC and a child without ECC.

It coincides with the results obtained by Hung *et al*. (2019) whose conducted a study with the aim of comparing machine learning methods in artificial intelligence to select the most relevant variables in the classification of the presence and absence of root caries and to evaluate the performance of the model, finding that of the machine learning algorithms evaluated, the SVM model demonstrated the best performance with an accuracy of 97.1%, a precision of 95.1%, a sensitivity of 99.6% and a specificity of 94.3% for the identification of root caries. The area under the curve for this model was 0.997. It also agrees with the results obtained by Sadegh *et al*. (2022) (20) whose found that the Multilayer Perceptron, Random Forest and Support Vector Machine predictive models performed the best in predicting caries occurrence with an accuracy value of 97.4%.

Contrary to the results found by Kang *et al*. (2022) (17) whose conducted a comparative study using four binary classification ML models (RF, GBDT, SVM and LR) on a dental caries dataset. After appropriate hyperparameter adjustment, RF showed the highest performance of 0.92, 0.90, 0.94 and 0.87 for accuracy, F1 score, precision and recall, respectively, in predicting the presence or absence of dental caries. Data collected from the 2018 children’s oral health survey conducted by the Korean Centre for Disease Control and Prevention under the Ministry of Health and Welfare were used. Also from Méndez *et al*. (2023) (29) whose found that the best model was the XGBoost model, with an area under the ROC curve (AUCROC) performance of 0.74 in population aged 1-17 years and 24 features, and a higher performance in the 1-5 years group, obtaining an AUCROC of 0.83 and 27 features, validated with data from the 2020 survey.

This discrepancy may be due to several reasons, such as the different risk factors considered for the analysis. Kang *et al*. (2022) (17) for example considered other factors such as the date of the last dental visit, hours of television viewing and the presence of caries in the study population. Similarly, in this study, age was not considered an essential characteristic as all children assessed were of the same age (12 years). Similarly, Méndez *et al*. (2023) (29) considered other factors such as lifestyle.

The similar values found between SVM and NN models are mainly due to the fact that both are able to consider non-linear solutions (although with different approaches) and that both work on the basis of parameters (although different in type and number). Both SVMs and NNs can address the same classification problem on the same dataset. This means that there is no reason arising from the characteristics of the problem to prefer one over the other (30,31).

The Orange Data Mining software, an open source program for data analysis that includes widgets with different prediction models, was used to perform the model comparison. It can be downloaded free of charge from http://orange.biolab.si. The main advantages of this program are: although it is based on the Python language, it is not necessary to use code to perform data analysis; the possibility of being able to observe the changes made in the procedures in real time; and it allows the generation of graphs.

The present study has some limitations, such as the fact that the sample comes from a specific population and therefore cannot be representative of other countries. Another limitation is the fact that the data set was limited (the sample size was only of 186 children) compared to other studies that have used a larger amount. The findings of the present study reveal that the use of machine learning models (Machine Learning) can help dentists in predicting factors associated with early childhood caries. However, more training data need to be added to achieve more stable and accurate results. Longitudinal studies are recommended to establish and confirm the predictive ability of the models. In conclusion, the best performing Machine Learning-based caries prediction models were Support Vector Machine (SVM) and Neural Networks (NN).

## Conclusions

The Machine Learning-based caries prediction models with the best performance were Support Vector Machine (SVM) and Neural Networks (NN). It is recommended to carry out longitudinal studies with a larger sample number to confirm the predictive capacity of the models.

## Figures and Tables

**Table 1:** Frequency of Early Childhood Caries (ECC).

	Frequency	Percentage	Accumulated
Without ECC	43	23.12	23.12
With ECC	143	76.88	100
Total	186	100	

**Table 2:** Performance indicators of the models evaluated.

Model	AUC	Accuracy	F1-Score	Precision	Recall	MCC
Logistic regression	0.911	0.909	0.938	0.905	0.974	0.774
Random Forest	0.854	0.891	0.927	0.884	0.974	0.728
Gradient Boosting	0.858	0.891	0.925	0.902	0.949	0.729
SVM	0.868	0.927	0.950	0.927	0.974	0.820
Neural Network	0.904	0.927	0.950	0.927	0.974	0.820
KNN	0.692	0.836	0.892	0.841	0.949	0.580

## Data Availability

The datasets used and/or analyzed during the current study are available from the corresponding author.
